# Emerging influence of RNA post-transcriptional modifications in the synovial homeostasis of rheumatoid arthritis

**DOI:** 10.3389/fimmu.2024.1494873

**Published:** 2024-12-09

**Authors:** Madiha Fatima, Fengmei Huang, Xiaohong Fu

**Affiliations:** ^1^ Department of Neurology, The Affiliated Yong-chuan Hospital of Chongqing Medical University, Chongqing, China; ^2^ State Key Laboratory of Neurobiology, The First Affiliated Hospital of Chongqing Medical University, Chongqing, China; ^3^ Medical Examination Center, The First Affiliated Hospital of Chongqing Medical University, Chongqing, China; ^4^ Central Laboratory of Yong-chuan Hospital, Chongqing Medical University, Chongqing, China

**Keywords:** RNA-post transcription modification, rheumatoid arthritis, synovial tissues, autoimmune diseases, RNA methylation

## Abstract

Rheumatoid arthritis (RA) is an important autoimmune disease that affects synovial tissues, accompanied by redness, pain, and swelling as main symptoms, which will limit the quality of daily life and even cause disability. Multiple coupling effects among the various cells in the synovial micro-environment modulate the poor progression and development of diseases. Respectively, synovium is the primary target tissue of inflammatory articular pathologies; synovial hyperplasia, and excessive accumulation of immune cells lead to joint remodelling and destroyed function. In general, epigenetic modification is an effective strategy to regulate dynamic balance of synovial homeostasis. Several typical post-transcriptional changes in cellular RNA can control the post-transcriptional modification of RNA structure. It can inhibit important processes, including degradation of RNA and nuclear translocation. Recent studies have found that RNA modification regulates the homeostasis of the synovial micro-environment and forms an intricate network in the “bone-cartilage-synovium” feedback loop. Aberrant regulation of RNA methylation triggers the pathological development of RA. Collectively, this review summarises recent advanced research about RNA modification in modulating synovial homeostasis by making close interaction among resident synovial macrophages, fibroblasts, T cells, and B cells, which could display the dramatic role of RNA modifications in RA pathophysiological process and perform the promising therapeutic target for treating RA.

## 1 Introduction

Rheumatoid arthritis is an inflammatory autoimmune disorder of the “bone-cartilage” unit, which may be developed as the symptoms of pain and disability, but its exact etiology remains mainly unknown (1). In the arthritic synovial microenvironment, the synovial intimate lining comprises fibroblast and macrophage like synovial cells. Together, they form an impermeable barrier. Meanwhile, fibroblasts, blood vessels, and interstitial macrophages are crucial components in the sublining layer. It is known to us that synovial homeostasis is shown to participate in the modulation of the progression of inflammatory arthritic diseases and aging-associated degenerative disorders (2, 3).

Synovial homeostasis is as dynamic as it is complex, which changes throughout the development and aging process during inflammation and disease. Once the synovial environment encounters signals such as trauma and inflammation, the macrophage barrier is lost (4). At the same time, the complex immunological modulatory network might be stimulated to make coupling interactions with synovial fibroblasts to initiate the remodelling and regeneration of synovitis and joint damage (5). In these procedures, various kinds of epigenetic patterns have been shown to participate in modulating the dynamic balance of the synovial microenvironment.

Recently, it has been shown that different kinds of RNA modifications regulate gene expression during the development or treatment of human diseases. As a key epigenetic modification in cellular biological processes, they could make functions via modulating the alteration of RNA structure (6, 7). In detail, RNA modifications are crucial in various immune cell formation, differentiation, activation, migration, and polarisation, which are among the basic processes that affect immune responses during tumour growth, inflammatory stress, and autoimmune illness (8).

Emerging evidence suggests that RNA modifications could maintain the dynamic balance of the synovial micro-environment by regulating the biological activities among various kinds of immunological cells (9). For example, the m^6^A methylation-mediated gene TGM2 alleviates the activity of anti-inflammatory drug sarsasapogenin in RA-FLS (10). The m^6^A change of ATG7 caused by METTL3 controls the autophagy-GATA4 axis and speeds up the ageing process and the development of arthritis (10). However, the impact of RNA modifications in synovial homeostasis regulations has yet to be explicated.

The current review focuses on the biological functions and underlying mechanisms of N6-methyladenosine (m6A), 5-methylcytosine (m5C), N1-methyladenosine (m1A), N7-methylguanosine (m7G), N4-acetylcytosine (ac4C), and pseudouridine (Ψ). It sheds light on the biological effects of RNA modifications on regulating synovial homeostasis and pathological arthritis. We also emphasise the therapeutic implications and potential functions of RNA alteration in early diagnosis of rheumatic diseases.

## 2 RNA modification

### 2.1 RNA m^6^A methylation

The m^6^A modification is methylation on N6 of adenine A, which occurs on RNA. Regarded as most prevalent post-transcriptional modification on eukaryotic mRNA and lncRNA, m^6^A mediates more than 80% of RNA methylation (6, 11). According to recent studies, some m6A marks might be added co-transcriptionally, which could enable RNA modifications to take place at the same time as transcription and affect RNA processing. The early regulatory checkpoint that this co-transcriptional addition may offer influences on RNA maturation and function before transcription is completed (12). Studies have shown that m^6^A modification performs vital functions in RNA translation, splicing, transport, stabilisation and localisation (11).

The modification of m^6^A is a reversible process, which affects the post-transcriptional fate of modified RNA. The deposition, elimination, and recognition of m6A modifications on RNA molecules are controlled by RNA-modifying enzymes, which include writers, erasers, and readers (**Table 1**).

**Table 1:** Characteristics and detailed information about several kinds of RNA modification.

RNA Modifications	Category	Regulators	Biological functions
M6A	Writer	METTL3/14/16, WTAP, VIRMA, HAIKA, ZC3H13, RBM15/15B	Regulates RNAs metabolism, degradation, translation, localisation, transcription Regulates snRNA pre-mRNA splicing Regulates pre-lncRNA splicing Regulates pri-miRNA processing
	Eraser	FTO, ALKBH5	
	Reader	YTHDF1-3, YTHDC1-2, IGF2BP1-3, HNRNPC/G/A2B1, eIF3, PRRC2A, SND1, FMR1, LRPPRC	
M1A	Writer	TRMT6/61A/61B,10B/10C, NML	Regulates translation Stabilises RNA structure Maintain ribosomal structure and function
	Eraser	ALKBH1/3/7, FTO	
	Reader	YTHDF1-3, YTHDC1	
M7G	Writer	METTL1, RNMT, WDR4, WBSCR22, TRM112	Regulates mRNA transcription, slicing, export, translation and degradation Regulates tRNA structure Promotes ribosome biogenesis Enhances miRNA processing
	Eraser	TSG1, H29K	
	Reader	eIF4E, CBC	
M5C	Writer	NSUN1-7, DNMT2	Modulates mRNA stability, export, translation Regulates tRNA structure and stability Affects miRNA maturation Increases lncRNA stability
	Eraser	ALKBH1, TETs	
	Reader	YTHDF2, ALYREEF, YBX1, FMRP	
ac4c	Writer	NAT10	Promotes mRNA stability and translation Promotes tRNA stability and maturation Boosts ribosome synthesis
	Eraser	N.A.	
	Reader	N.A.	
pseudouridine (Ψ)	Writer	DKC1, PUS1/3/7/10, TRUB1/2, RPUSD3/4, H/ACA snoRNPs	Affects mRNA translation Maintain tRNA structure and Controls rRNA folding Influence pre-mRNA splicing
	Eraser	N.A.	
	Reader	N.A.	

Methyltransferases that catalyse the m6A modification of adenosine on mRNA include METTL3/14, WTAP, and KIAA1429 [17]. METTL3/14 and WTAP form a methyltransferase complex (MTC). Among them, METTL3 and METTL14 constitute the main components of MTC and perform the function of m6A deposition on nuclear RNAs. The WTAP mechanism guarantees the specific distribution of the METTL3-METTL14 complex to nuclear speckles and preserves its catalytic activity towards mRNA targets. Except for the canonical writers, MTC accessory subunits have a significant impact on the catalytic process include RBM15, ZC3H13, VIRMA, and HAKAI (12).

Demethylases, including two enzymes such as FTO and ALKBH5 (both belonging to the ALKB family), removed the methylated base that has undergone m^6^A modification. In detail, Previous studies such as Chuan He’s research group discovered FTO as the first m^6^A demethylase in 2011, and it primarily facilitates mRNA and snRNA demethylation at m^6^Am residues (13). But recent studies have explained that FTO is the first RNA demethylase that mediates the demethylation of m^6^A (!4). In 2013, ALKBH5 was discovered as another demethylase, which combines with specific m^6^A-modified single-stranded RNA to carry out the demethylation of m^6^A (14). In addition, Xie et al. have introduced flavin mononucleotide (FMN) as a new synthetic molecule derived from vitamin B2, plays a crucial role in enzymatic reactions (15).

The primary function of reader proteins is specifically recognised RNA that binds to the m^6^A modification, activating downstream regulatory pathways. YTH domain protein is well known as m^6^A recognition protein. YTHDF1 and YTHDF3 are mainly used to enhance the efficiency of mRNA translation, while YTHDF2 has been found to mediate RNA degradation (16, 17). Except for these direct readers, three heterogeneous nuclear ribonucleoproteins (hnRNPs) indirectly associate with m^6^A sites according to the “m^6^A switch” mechanism. Dysfunction of enzymes related to m^6^A modification may result in several autoimmunological diseases, such as rheumatoid arthritis and other musculoskeletal disorders (18). These m^6^A sites preferentially occur in the relatively conservative RRACH sequence (R represents A/G, H represents A/C/U), displays at the 3’-UTR, termination codon, and long exons of mRNAs (6).

Experiments have shown that m^6^A controls the dynamic equilibrium of the bone matrix and establishes an intricate network in bone metabolism (19). The m6A alteration at the cellular level modulates the expression of ALP, Runx2, Osterix, VEGF, and other associated genes, influencing physiological processes. m6A is involved in the delicate procedures of adipogenesis and osteogenesis from MSCs, which is important for bone homeostasis (19, 20).

### 2.2 RNA m^1^A methylation

m^1^A methylation is another key post-transcriptional modification in RNA modification, identified by Dunn in the 1960s, which occurs on N1 adenosine (21). It is well-established that including this methyl group disrupts the specificity of base-pairing, revealing the regulatory roles of this alteration in RNA molecules. Multiple investigations have evidenced the extensive presence of m^1^A in mRNA, tRNA, rRNA, and mitochondrial transcripts (22, 23).

According to Dominissini et al., m^1^A is abundant in the 5’-UTR of mRNA. It tends to present at the initiating codon upstream of the first splicing site, which may influence the process of mRNA translation (24). Besides, m^1^A methylation may display the promising capability to negatively regulate reverse transcription and make changes according to relevant stimulation in this microenvironment (25). m^1^A is inextricably linked with m^6^A modification because m1A can not only rearrange to m^6^A (Dimroth rearrangement) under alkaline conditions but also share some regulators with this modification (26). Like m^6^A methylation, these enzymes of m^1^A, including “writer” (TRMT10C/61B, TRMT6/61A), “eraser” (ALKBH1, ALKBH3), and “reader” (YTHDF1-3, and YTHDC1), exert their effects in the post-transcriptional stage of mRNA and ncRNAs. YTHDF1-3 and YTHDC1 are regulatory factors that have participated in the detailed procedures for m^1^A and m^6^A (27). Respectively, the deeply advanced research about the regulation of m^6^A may be supported to investigate the mechanism of m^1^A further (28).

Moreover, recent studies have illustrated that m^1^A affects RNA base pairing, thereby influencing the structure and function of target RNA molecules. Human rRNAs and tRNAs contain numerous diverse m^1^A modification sites, such as position 1322 of 28S rRNA, position 947, and position 58 of tRNA (26). In mRNA, m^1^A occurs in each segment, including CDS, 5’-UTR, 3’-UTR performs effective capabilities according to the different regions or subcellular locations (28, 29).

### 2.3 RNA m^7^G methylation

m^7^G, the RNA methylation of guanine at the N7 position, accounts for about 0.4% of all guanosines. As a well-known 5’ cap (m7GPPPN) modification of mRNA, m^7^G regulates mRNA splicing and nuclear export and contributes to mRNA translation (30). m^7^G modification is like m^1^A in modification level and widely exists in tRNA (at position 46), 18s rRNA, mature and even pre-miRNAs (31), which is accumulated in transcript fragments of mRNA 5’-UTR, CDS, and 3’-UTR, pre-mRNAs (32).

In m^7^G, METTL1, RNA (guanine-7-) methyltransferase (RNMT), WD repeat domain 4 (WDR4) complex, and Williams-Beuren syndrome chromosomal region 22 protein (WBSCR22) are recognised as components involved in its modification (33). Among them, RNMT is necessary for effective cap methylation and is activated by the mini protein (RAM). For most non-coding RNAs, the demethylation process of the m^7^G cap can be completed by cleavage or further modification to m^2,2,7^G trimethyl guanosine. The recognition of m7G cap is mostly performed through eIF4E and cap-binding complex (CBC), therefore, regulating RNA maturation, nuclear export, and translation (34, 35). Interestingly, the working mechanism of METTL1 and WDR4-modified tRNAs has been further explored by two research teams and reached consistent and complementary conclusions, revealing that in addition to the N-terminal of METTL1, the C-terminal of WDR4 is also very important for normal activity of METTL1, and found that the N-terminal of METTL1 participates in the construction of active pockets, and inhibits its methyltransferase activity through the steric hindrance effect triggered by phosphorylation (36).

m^7^G is present in mRNA and is crucial in orchestrating translation procedures. Moreover, it exerts considerable influence on stem cell proliferation and differentiation (37).

### 2.4 RNA m^5^C methylation

An alteration known as RNA m^5^C was initially discovered in 1958, involving the methylation of the fifth carbon atom of RNA cytosine. m^5^C methylation occurs on every RNA molecule, such as tRNA, rRNA, mRNA, and ncRNA, which are key molecules to maintain the regular physiological functions of eukaryotic cells (29, 38).

It enhances RNA stability and regulates protein synthesis and translation. According to Amort et al., m^5^C is primarily located at mRNA’s 5’ and 3’-UTRs, particularly close to the translation start codon. It increases the stability of RNA and controls the processes of protein synthesis and translation (39). m5C can stabilise tRNAs by enhancing the thermal stability of hydrogen bonds with guanine, providing protection against inappropriate degradation. Meanwhile, the m^5^C residue of rRNA mostly regulates the quality of ribosome biogenesis (40).

RNA m^5^C was also monitored by “writers”, “erasers”, and “readers” as presented in **Table 1**. m^5^C methyltransferases (“writers”), including NSUN1-7 and DNA methyltransferase-like 2 (DNMT2) (41, 42). Specific m^5^C writers catalyse different RNA subsets. TET proteins catalyse the removal process (TET1-3) and ALKBH1, mainly oxidising m^5^C to 5-hydroxymethylcytidine (hm5C) (38). In all cases, the formation of hm5C will reduce the modification of m^5^C, so TETs and ALKBH1 are identified as erasers (40). The Aly/REF nuclear export factor (ALYREF) is the first identified reader, which can bind to m^5^C sites to perform biological functions (43). Dominissini et al. confirmed that ALYREF facilitates the nuclear export of mRNAs. Subsequently, ELAVL1 and FMRP were also identified, which could directly bind to m^5^C to modulate the distribution of m^5^C in mRNA and affect the maturation of rRNA by regulating the m^5^C levels (44). The level of m^5^C is closely related to immune diseases.

### 2.5 RNA N4-acetylcytidine

ac4C is the RNA acetylation of cytosine at N4 position, which occurs in cytidine conservatively. It is the only acetylation modification that has been studied in eukaryotic RNA. Like many RNA modifications, ac4C was first detected in tRNA, rRNA, and mRNA (45). So far, only n-acetyltransferase 10 (NAT10) is considered the writer of ac4C, an indispensable ATP-dependent RNA acetyltransferase (46, 47). It can catalyse ac4C modification in multiple RNAs, for example, 18S rRNA, tRNA and mRNA. During the formation of ac4C, two additional proteins, including C/D snoRNA U13 and THUMPD1, are required to act together in the catalytic process (48).

As a newly discovered RNA modification, ac4C is still unknown, especially its regulator and molecular function. Only one writer, eraser, and reader has yet to be found. The presence of ac4C in the mRNA CDS region can significantly improve mRNA stability and promote protein translation, especially in the translation process, which may influence the interaction with homologous tRNAs (42). In 18sRNAs, ac4C plays a crucial role in pre-rRNA processing and ribosome production, directly impacting translation capability. On the other hand, ac4C can mainly promote the stability of tRNA and serve as a monitoring indicator for the maturation of eukaryotic tRNA (47–49).

### 2.6 Pseudouridine (Ψ)

Pyrimidine nucleoside is formed by linking Ψ, a trace nucleic acid base, located at the fifth position of uracil and ribose. Ψ is connected to ribose base with covalent bonding. One of the post-transcriptional modifications, Ψ, is widespread in RNAs, particularly in long non-coding RNA (lncRNA), where it enhances the activity of tRNA and rRNA by fortifying the structure of the RNA chains (50). The studies have reported about pseudouridine synthase 1 (DKC1) count and RNA-independent single pseudouridine synthetases (PUSs) comprising PUS1/3/7/10, PUSL1/7, TRUB1/2, and RPUSD1-4 that led to distinct cellular localisation and RNA targets (51).

**Figure 1:**
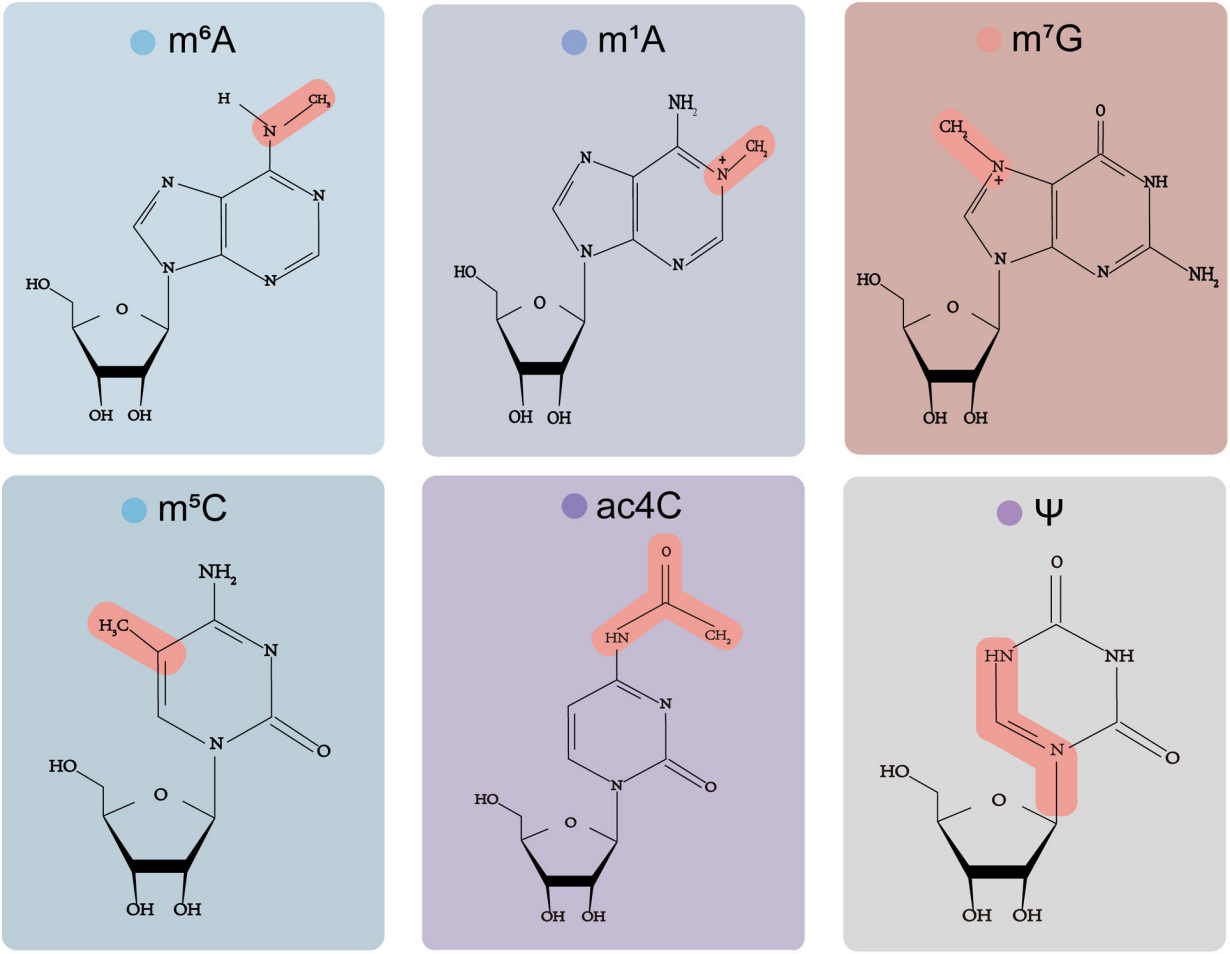
Chemical structures of six RNA modifications.

Researchers have demonstrated that Ψ enormously influences RNA’s biogenesis, structure, stability, and function and regulates gene expression. There are many pseudouridine sites in tRNA, which are crucial for the stability of the structure and mediate the base pairing of tRNA codon-anticodon, thus affecting the translation process (50). Moreover, Ψ regulates the pre-mRNA stage through various functional modes, such as mRNA/rRNA/snRNA structure, stability, translation, and termination (49). The chemical structure of all six modifications are showed in **Figure 1**.

## 3 Synovial homeostasis

The environment of the synovial joint presents immunological challenges. Under conditions of stability, specific groups of immune-regulatory cells (such as FLS, macrophages, T and B cells, etc.), consistently inhibit inflammation in the synovial joint in reaction to harmful signals from cartilage and synovial fluid, which build up under mechanical compression. In the presence of inflammation, the immune-regulatory environment is disrupted, leading to the involvement of activated immune cells in synovial inflammation and joint erosion. Interacting, these cells generate numerous pro-inflammatory cytokines, vascular endothelial growth factor (VEGF), and other mediators that penetrate the joint cavity, therefore facilitating the pathological progression of articular disease.

Scholarly investigations have shown that the maintenance of synovial homeostasis relies on the equilibrium between the proliferation and cell death of FLS. Ferroptosis was enhanced by glycine through SAM-mediated methylation of the GPX4 promoter in rheumatoid arthritis, while ferritin reduced the alterations in FLS between proliferation and death (48). There is compelling evidence that Notch signalling is critical for cell survival and synovial homeostasis (43). The Notch signalling pathway regulates cell development and differentiation that plays an inflammatory driving role. Notch receptors are expressed and activated in synovial cells, and Notch signalling promotes the progression of arthrophlogosis by regulating the differentiation and function of various immune cells and stromal cells.

Furthermore, synovial macrophages have been identified as key regulators in maintaining the balance of synovial tissue, which in turn determines the development and advancement of inflammatory joint disorders (3). Under homeostatic conditions, macrophages from diverse sources become tissue-resident macrophages in the synovial micro-environment, maintaining their quantity and displaying anti-inflammatory properties. Their absence of pro-inflammatory gene expression enables them to promptly and efficiently detect the danger signals of apoptotic cells and sources of damage, therefore averting spontaneous inflammatory responses triggered by damage in healthy tissue micro-environments (52). In contrast to synovial homeostasis, macrophages from circulating blood monocytes continuously invade inflammatory tissues and undergo differentiation into newly recruited macrophages under various inflammatory conditions. This process involves the expression of pro-inflammatory genes, so contributing to the host defence and inflammatory response.

The independent Nature Medicine articles authored by Saito et al. and Yang et al. have unveiled a novel insight into the migration of HIFs throughout the synovial joint. A key function of the transcription factor HIF-2α in maintaining synovial homeostasis was elucidated. It was shown that increased activity of HIF-2α caused by stress overshadows the favourable effects of the closely related HIF-1α, promoting the breakdown of joint synovial homeostasis (53, 54). During normal, non-stressed conditions, the hypoxia response transcription factor HIF-1α maintains stable joint function, which includes the normal synthesis of cartilage extracellular matrix and the promotion of autophagy. Contrarily, HIF-2α arranges itself in heterodimers with ARNTL and employs complementary pathways (IHH and RUNX2) to directly stimulate the expression of certain members of the MMP family. Hence, it facilitates the advancement of inflammatory joint disorders.

Indeed, the involvement of inflammatory cytokines in the onset and progression of joint disorders is widely acknowledged, their function in maintaining and disturbing synovial homeostasis in joints should not be disregarded. Synovial cells are passive victims of cytokine destructive forces and active participants in maintaining cytokine control to maintain joint integrity. Research has shown that balancing cytokines and anabolic growth factors in healthy articular cartilage is crucial for maintaining appropriate tissue homeostasis.

## 4 RNA modification in synovial homeostasis

**Figure 2:**
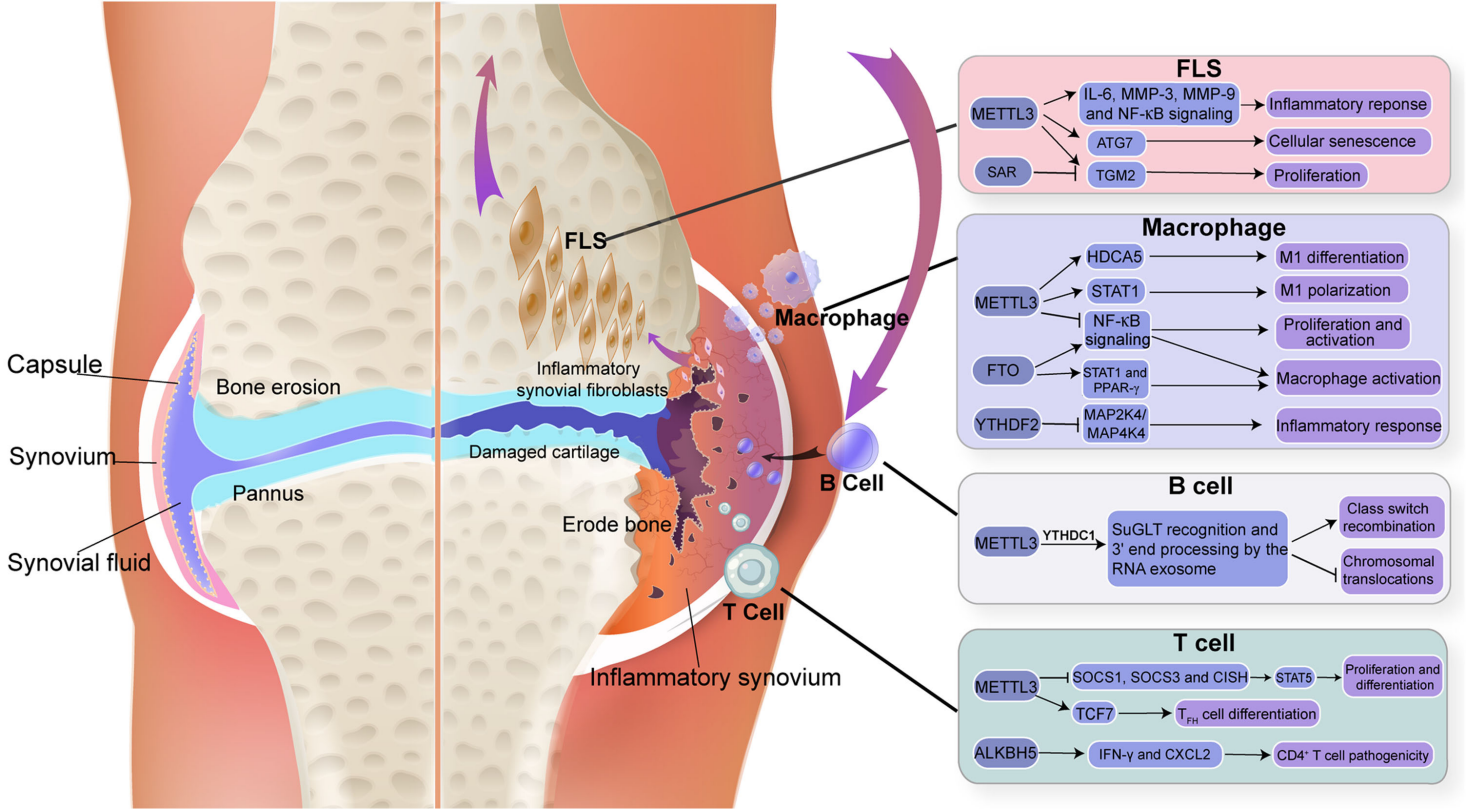
Triggering modifications in RNA component and immune cells of synovium. The figure depicted m^6^A modification in RNA, which is important in regulating immune function in synovial homeostasis.

As a kind of gene regulation at the post-transcriptional level, RNA modification is precisely regulated by various enzymes and participates in all aspects of RNA metabolism, thus involving diverse biological processes such as development, metabolism, immunity regulation, cellular differentiation, bone homeostasis, etc. Disruption of RNA modification results in impaired gene expression and cellular function, triggering the interaction changes among fibroblast synoviocytes, macrophages, T cells, and B cells in the synovial microenvironment homeostasis (43). Many studies have revealed the main biological functions of RNA modification on various immune cells in synovial homeostasis, as described below (**Figure 2**).

### 4.1 RNA modification and fibroblast synoviocytes

**Figure 3:**
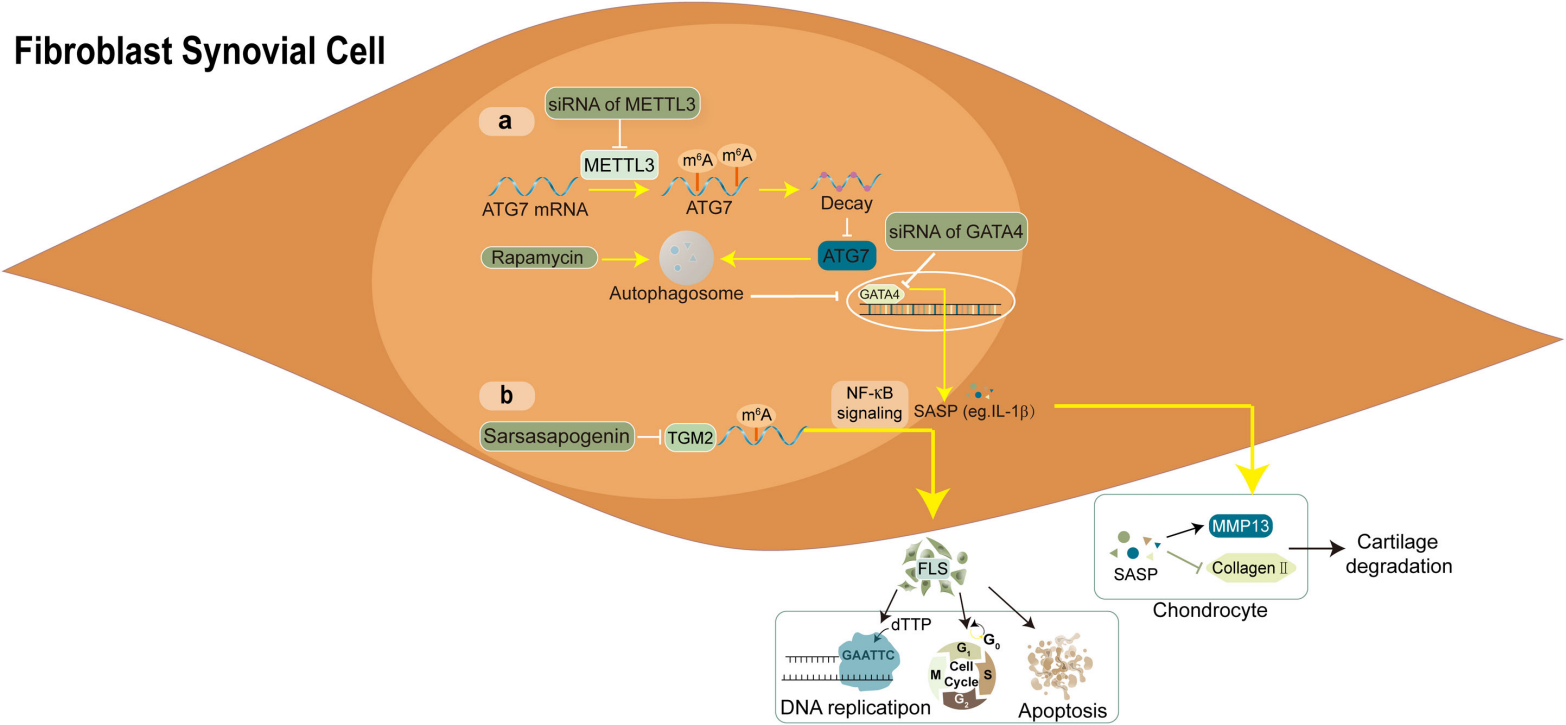
Molecular mechanism and RNA modifications with their functions in FLS. **(A)** the autophagy-GATA4 axis is regulated by METTL3-mediated m6A modification of ATG7, which accelerates the onset of arthritis and cellular senescence. **(B)** the sarsasapogenin’s inhibitory action in FLS is partly attributed to the N6-methyladenosine modification of TGM2 mRNA.

FLS are highly specialised mesenchymal cells in the synovium of joints, mainly in the intimal lining layer. FLS is the core target cell of RA, which could switch from early immunosuppressive to stimulatory in response to relevant factors (55). During normal physiological conditions, the intimal lining forms a thin and permeable barrier at the boundary between the sublining and the synovial fluid canal. Synovial fluid synthesis (FLS) regulates the composition of the extracellular matrix (ECM) and synovial fluid, providing lubrication and nourishment to the cartilage surface (56, 57). Therefore, as significant effector cells, FLS have special aggressive behaviours and function actively in the pathogenesis and progression of RA. **Figure 3** illustrates the main RNA modification in FLS.

Jiang and colleagues established the m6A transcriptional map in human rheumatoid arthritis fibroblast-like synovial cell line MH7A cells using m^6^A seq and RNA seq. The potential relationship between m^6^A and FLS in RA is described for the first time (58). Compared with the healthy controls (HCs), single-cell analysis and machine learning efficiently predicted that IGFBP2 was elevated and METTL3 was downregulated in RA-FLS. By searching the GEO database, four different data sets showed the increased expression of the IGF2BP3 gene in RA synovial cells. Moreover, molecular docking and *in vitro* experiments suggested that IGF2BP3 is also a target gene of triptolide (TP), which has been proven to have therapeutic potential for RA.

METTL3, a crucial component of the m6A methyltransferase complex, has been confirmed to promote activation and inflammation of FLS through the NF-κB signalling pathway in RA (59). Furthermore, Shi et al. demonstrated that METTL3 reduced autophagy activity by weakening the stability of autophagy-related 7 (ATG7) mRNA in a manner dependent on m6A-YTHDF2. This, in turn, facilitated the ageing of FLS and the progression of arthritis (60). In addition, Lin et al. identified an m^6^A methylation-mediated gene TGM2 and portrayed the regulatory mechanism in controlling the immune microenvironment of RA-FLS (10). This cross-validated experiment verified the role of m^6^A RNA methylation in synovium and identified a novel target for RA treatment.

Although little is known about other RNA modifications that regulate FLS cells in the synovium microenvironment, previous literature has confirmed that RNA modification is closely related to RA-FLS biology. **Figure 3** illustrates the main RNA modification in FLS.

### 4.2 RNA modification and macrophages

**Figure 4:**
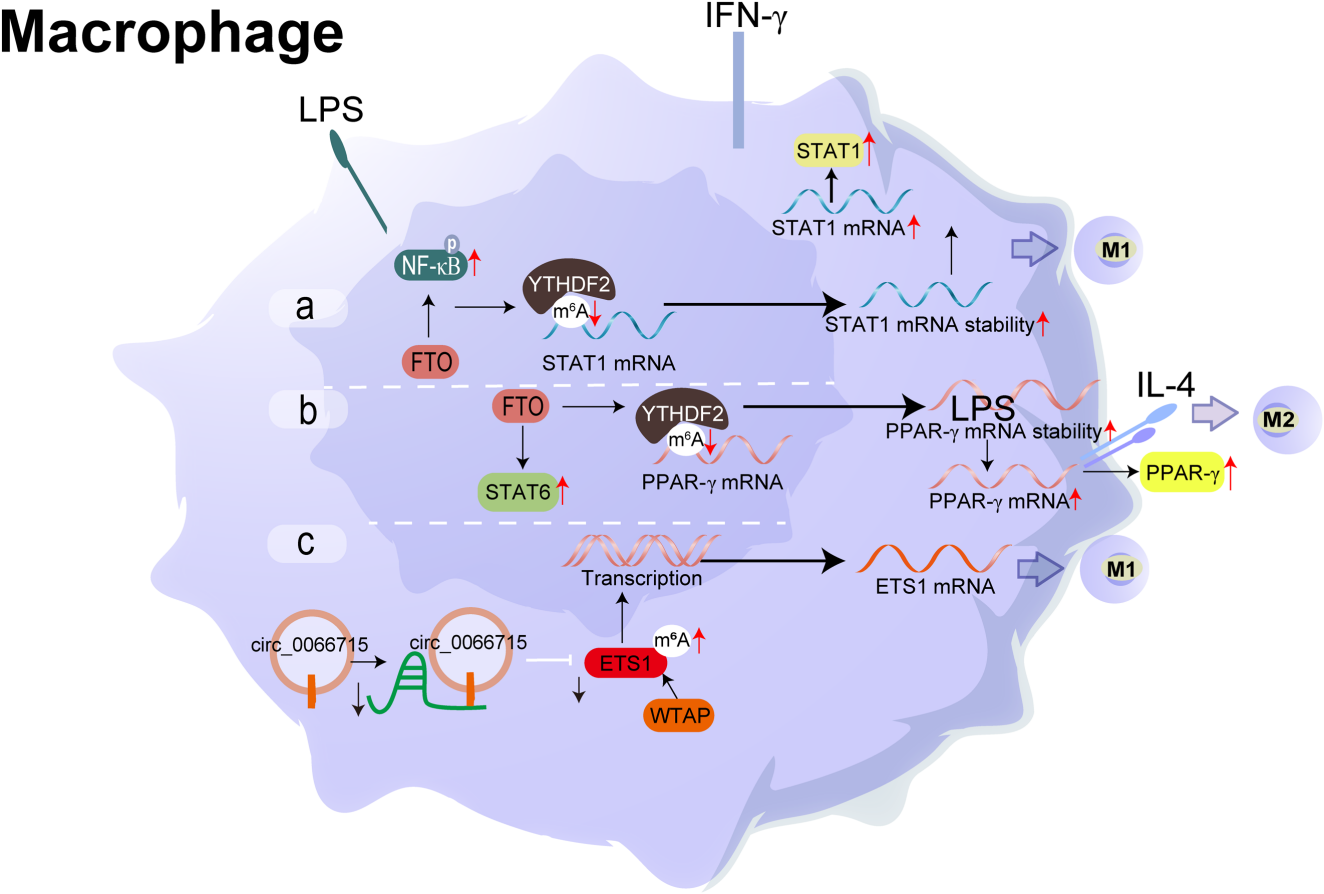
Molecular mechanism and RNA modification with their functions in Macrophage. **(A)** FTO activates the NF-κB signalling pathway, boosting the mRNA stability of STAT1 and PPAR-γ through YTHDF2 participation, thereby promoting M1 macrophage activation. **(B)** FTO increases the mRNA stability of STAT1 and PPAR-γ, promoting M2 macrophage activation. **(C)** circ_0066715 up-regulated the expression of the target gene ETS1 and exerted a miRNA sponge-like impact of miR-486-5p. WTAP also contributes to the promotion of the expression of ETS1. m6A regulatory mechanism and the circRNA/miRNA/mRNA regulatory axis are involved in the polarisation process of RA macrophages.

The synovial lining and sublining layers contain numerous HLA-DR+ macrophages in the synovium, which are activated through antigen presentation (61). Generally, synovium macrophages are composed of two types, tissue-resident macrophages and circulating macrophages. New evidence suggests that the cellular function of macrophages varies from the different subsets of macrophages in synovial homeostasis (62). These macrophages are potent cytokine producers with apparent activated status and professional antigen-presenting cells (APCs) that stimulate an adaptive immune response. After that, T lymphocytes were activated and largely promoted joint destruction. T lymphocytes at synovium and pan membranous cartilage junction increased significantly. Abundant-activated macrophages are an early hallmark of arthropathies, and many macrophages have become a significant feature of inflammatory lesions (63). The degree of synovial macrophage infiltration is correlated with the degree of joint erosion and depletion of these macrophages from inflamed tissue (64). This phenomenon also has a profound therapeutic benefit. Importantly, there is bidirectional intercellular communication between macrophages and FLS in the arthritis synovium, which is beneficial in governing the maintenance or disruption of joint homeostasis (**Figure 4**).

Macrophages M1/M2 polarisation is mainly mediated immune/inflammatory response and maintained the dynamic balance in synovium (65). Classical theory presumed that M1 (pro-inflammatory) macrophages are activated by interferon-γ, lipopolysaccharide (LPS), or tumour necrosis factor-alpha (TNF-α). Meanwhile, it secretes large amounts of proinflammatory cytokines and mediators, such as TNF-α, interleukin-1/6/12 (IL-1/6/12), and cyclooxygenase-2 (COX-2). M2 (anti-inflammatory) macrophages termed as wound-healing macrophages, are polarised by the Th2 cytokines, IL-4 and IL-13. These are further classified into specific subtypes. M2 macrophages shows an anti-inflammatory phenotype, which is helpful for tissue repair and remodelling (66). However, M1/M2 can’t be regarded as the extreme of the activation state spectrum of macrophages, which is just a point reflecting the complex environment of the synovium. While the imbalance of M1/M2 macrophages, correlated significantly positively with synovial homeostasis (67). A complex cytokine and chemokine network is involved in the inflammatory environment.

Recently, accumulated evidence has revealed the potential links between m^6^A modification and macrophage polarisation. METTL3, the writer of m^6^A, facilitates M1 macrophage polarisation through the methylation of STAT1 mRNA directly and attenuates M2 macrophage polarisation (68). While the reader of m^6^A (69), FTO strengthened the polarisation of M1 and M2 macrophages, FTO activated the NF- κB signal path, and increased the mRNA stability of STAT1 and PPAR-γ through YTHDF2 involvement, thereby accelerating macrophage activation (70). Combined with methylated RNA immunoprecipitation (MeRIP seq) and RNA sequencing results, Wan et al. evaluated the whole transcriptome m6A modification in the synovium of RA patients. They showed that M1 macrophages in RA patients were significantly increased (71).

Wu et al. found that YTHDF3 (m^1^A reader) may participate in the polarisation of M1 macrophages by regulating a series of target genes (72). Utilising the Lasso regression analysis, researchers constructed a novel m^7^G risk signature or m^5^C prognostic model in both hepatocellular carcinoma and prostate cancer (73–75). They found a significant correlation between the expression level of m^5^C regulator genes (NSUN2/6, TET1/3) and immune cells, including M1/M2 macrophage infiltration characteristics (76, 77). Similarly, consistent with the previous results, Aly/REF and NUSN2/3 (m^5^C modulators) interacted with lncRNA to trigger the occurrence of disease, which shed new light on the role of m^5^C modification within the process of immune infiltration of macrophages (74). NAT10, as a critical gene regulating the formation of N4-acetylcytidine in RNA, has a significant positive correlation between immune infiltration (75), including B cells, CD8^+^T cells, CD4^+^T cells, neutrophils, macrophages, and other immune cells (69). Seven writers of the pseudouridine (Ψ) modification were significantly enriched in the immune-related signal pathway of macrophages (78). Unfortunately, these studies were carried out in other diseases, and there is no research report on RNA modification except m^6^A in synovium.

### 4.3 RNA modification and T cells

**Figure 5:**
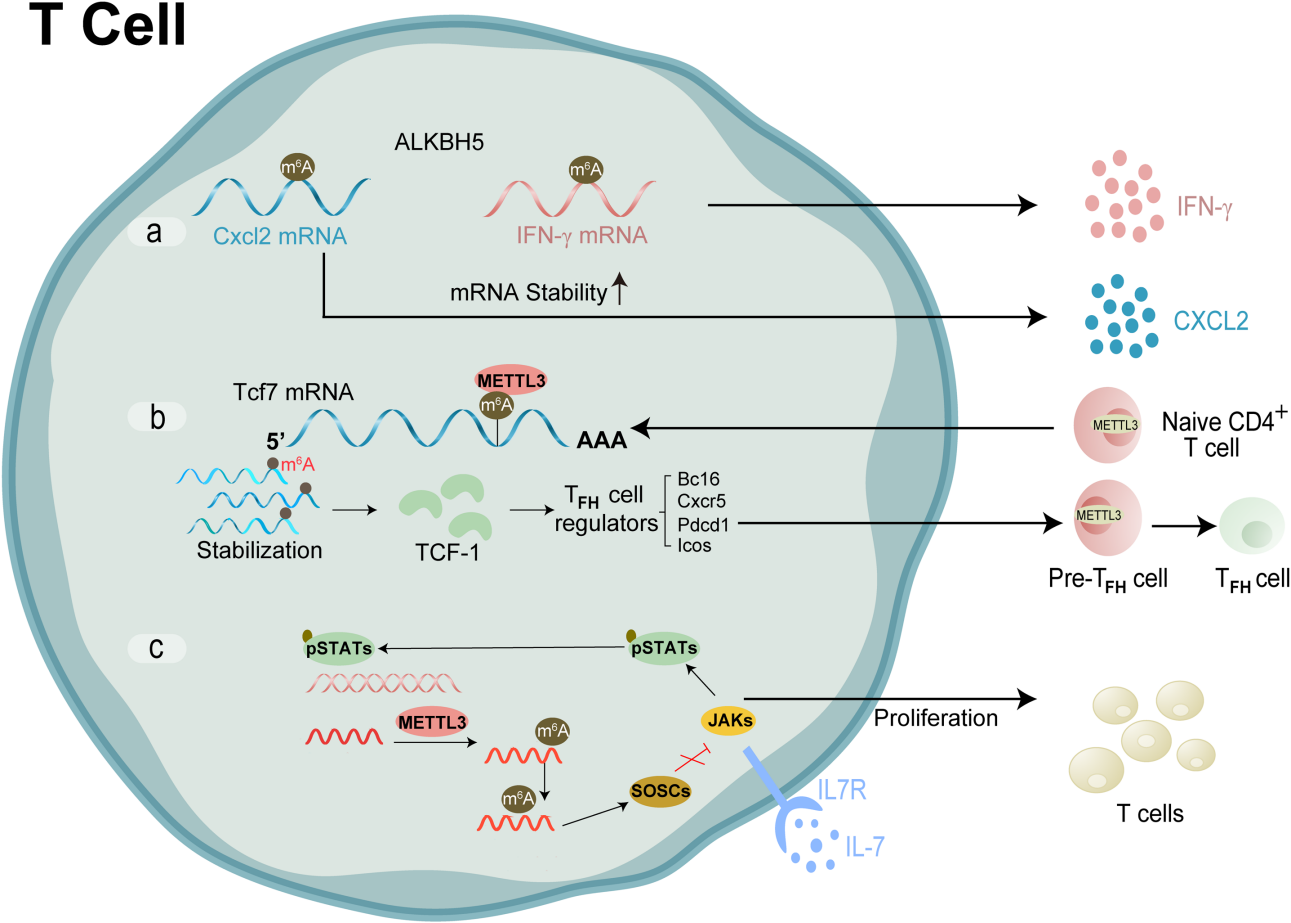
Molecular mechanism and RNA modification with their functions in T cell. **(A)** ALKBH5 reduced m6A modification on interferon-γ and CXCL2 mRNA, thus enhanced their mRNA stability and protein expression in CD4+ T cells. **(B)** METTL3 modifies the m6A gene to increase the Tcf7 transcripts. TCF-1 controls Tfh cell expressions, which in turn triggers the Tfh transcription program and further encourages the transition of pre-Tfh cells into Tfh cells **(C)** m6A lead SOCSs to undergo rapid mRNA degradation upon IL-7 stimulation, enables IL-7-JAK signalling to activate downstream phosphorylation STATs, which in turn starts the reprogramming of naïve T cells for proliferation and differentiation.

**Figure 6:**
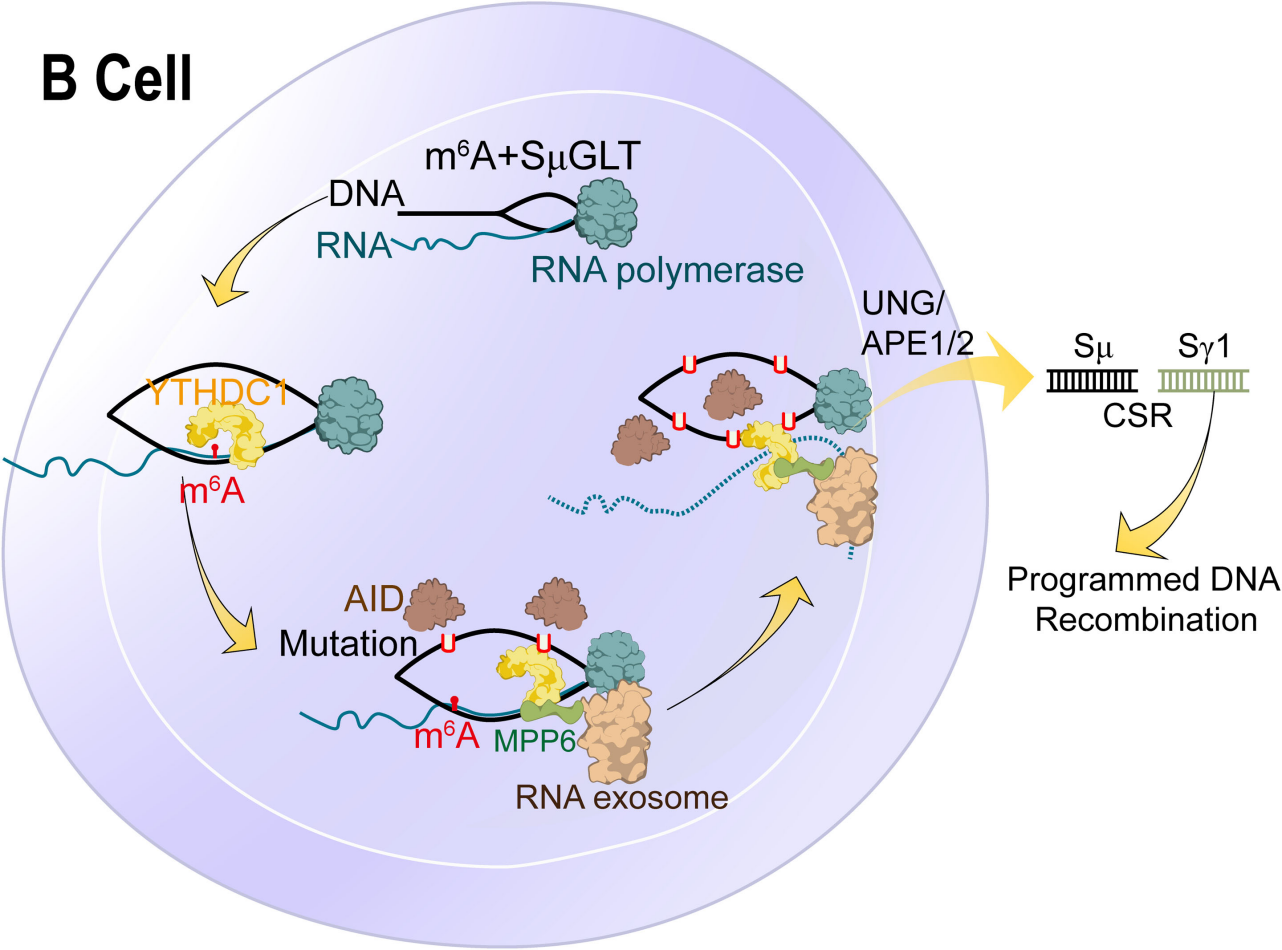
Molecular mechanism and RNA modification with their functions in B cells. N6-methyladenosine (m6A) RNA modification, which is catalysed by the METTL3 enzyme, is responsible for the recognition and 3’-end processing of SμGLT by the RNA exosome. This mechanism promotes class switch recombination (CSR) and suppresses chromosomal translocations.

Specific T cells originate from pluripotent stem cells in the bone marrow, mature in the thymus, and then trafficked to the inflammatory sites, developing into the pathophysiology of synovitis (79, 80). T cells are transferred from the bloodstream to the synovial tissue through interactions with the endothelial cells in the posterior venules of synovial capillaries (73). Then, many immune cells infiltrate, including macrophages, granulocytes and B cells, especially CD4^+^ T and CD8^+^ T cells, increasing the expression of adhesion molecules and chemokines in the endothelium, thus activating a systemic immune response and forming a local synovium. It has been proved that CD4^+^ T cells, rather than CD8^+^ T cells, initiated the synovial inflammation process, the hyperactivation of the immune response, and the presence of autoantibodies in the synovial microenvironment is sufficient to trigger the development of rheumatic diseases (74, 75). **Figure 5** presents the machinery of RNA modifications and their molecular function in T cells (**Figure 6**).

Among them, CD4^+^ T cells infiltrated in synovial joints could be subdivided into several subsets based on their differential effector functions. For instance, TH1 subsets defended against intracellular pathogens and activated macrophages. TH1/Tfh/T helper-like can sustain the B cell-Ig switch, while the primary function of TH17 was to recruit neutrophils and induce proinflammatory factors. Cytotoxic CD4^+^ T cells mediated cell death and participated in the hypercitrullination of Neutrophil Extracellular Traps through a perforin-dependent mechanism (81). Parallel to the characterisation and classification of T cell subsets, the contribution of various effector T cells to synovial homeostasis has been investigated in numerous studies, although sometimes with contradictory results. Reduced functions and frequencies of Treg cells contribute to the pathogenesis of RA (82). m^6^A plays a significant role in maintaining T cells’ homeostasis and differentiation stability. Zhao group established a nomogram based on the immune cell model employing GEO databases and cluster analysis (83). Finally, CLP1 was highly correlated with Tfh infiltration, which may play a non-negligible role in the synovium via altering immune cell infiltration. It could be a novel target for clinical diagnosis and therapy of joint disorders (84).

Luo et al., 2021 has described the role of m6A as a prevalent internal mRNA modification that regulates various physiological functions and disease progression, specifically emphasising its involvement in chronic inflammation, which is a hallmark of rheumatoid arthritis. They argue that emerging evidence points to m6A methylation as a significant player in inflammatory responses, suggesting that understanding this modification could provide insights into the pathogenesis of inflammation-related diseases (85).

Xu et al., have highlighted that m6A modifications not only regulate post-transcriptional processes but also influence cellular responses, including necrosis, apoptosis, and autophagy, which are critical in the context of inflammatory diseases. Their findings indicate that m6A is integral to the regulation of inflammation, thereby reinforcing the notion that m6A dysregulation may contribute to the development of conditions such as rheumatoid arthritis (86).

Huang et al., have described the myth about m6A methylation that is intertwined with fibrosis and inflammation regulation, particularly through the functional dynamics of m6A methyltransferases, demethylases, and binding proteins. The authors posit that disruptions in this network can lead to abnormal m6A levels, resulting in altered RNA expression and the onset of autoimmune disorders. They also note that m6A modification is crucial for innate immune responses, suggesting that m6A dysfunction could be a key factor in the pathophysiology of autoimmune diseases like rheumatoid arthritis (87). The crosstalk of RNA modification regulators and the potential roles in T cell infiltration and tumor microenvironment rheumatoid arthritis have been widely researched. Zhou Jing’s team has confirmed that METTL3 produces a marked effect on T cells (88). It is investigated that in condition of rheumatoid arthritis METTL3 governs the homeostasis and differentiation of T cells by targeting the IL-7/STAT5/SOCS signal axis and regulates the stability of Treg cells via targeting the IL-2/STAT5/SOCS pathway (89). Likewise, Yao et al. showed conditional deletion of METTL3 in CD4^+^ T cells impaired Tfh differentiation and germinal center responses in a cell-intrinsic manner in mice (90). Subsequently, their research group confirmed that m6A eraser ALKBH5, but not FTO, maintains the ability of naïve CD4^+^ T cells to induce adoptive transfer colitis, which resulted in attenuated CD4^+^ T cells responses and diminished recruitment of neutrophils during T cell-mediated inflammation and autoimmunity (88). RNMT (m^7^Gcap methyltransferase), under the action of m^7^Gcap binding protein LARP1, promotes T cell activation by increasing ribosome abundance (91).

Guo et al. identified 455 m^5^c and 26 ac4C methylation transcripts by RIP-Seq and mass spectrometry in CD4^+^T cells (84). The methylation expression profile analysis found that NSUN2, as the major m^5^C methyltransferase, significantly decreased its expression in CD4^+^T cells. These ac4C-related dysregulated transcripts mainly monitor mRNA’s catabolic process and translation initiation (92). Besides, m^7^G modification level is closely related to the immune infiltration characteristics in patients with COVID-19. The expression of m^7^G-related genes in COVID-19 patients was higher than in non-COVID-19 patients (93).

### 4.4 RNA modification and B cells

Both synovial and intra-articular B cells are recruited from the peripheral blood to inflammation sites, driven by CC and CXC chemokines that express receptors on B lymphocytes (94). Therefore, synovial tissue becomes the active area for B cell aggregation, proliferation, plasma cell differentiation and autoantibody production. Synovial B cells are usually located in the T-B cell region and maintained under sustained stimulation by cytokines, including proliferation-inducing ligand (APRIL) and lymphocyte stimulator (BLyS) (95). Currently, studies have found that in blood and synovium of patients with RA, memory (CD27+), B lymphocytes (including swIg and LM B lymphocytes) increased, accompanied by an activated (CD95+CD21 low) phenotype (96). **Figure 6** explains the molecular mechanism and RNA modification with their functions in B cells.

B cells secreted substantial proinflammatory cytokines under the control of transcription factor NF-kB. In the early and late stages of arthritis, activated NF-kB is generally present in B cells and other cells (including macrophages, T cells, and fibroblast synoviocytes) in synovial tissue (97). B lymphocytes are conducted to antigen presentation and immune complex formation, then internalised by dendritic cells and macrophages through an FCγR-mediated process (98). This conclusion has been verified in a murine model of autoimmune arthritis, in which both T cells (CD4+) and antigen-presenting cells jointly promote the occurrence and development of the disease. Still, the premise is that B-cell or IgM-deficient mice will not develop arthritis even under the condition of immunity (92).

Previous studies have illustrated that activated B cells release cytokines to interact with T cells and neutrophils (99). Stimulated T cells react to other chemokines and guide them to search for their B-cell partners. The migration route of B cells with sufficient antigen binding from the naive B cells was redirected and moved to the boundary of B cells and T cells. Similarly, B cells receive the help of T cells, which assist in forming autoantibodies in an HLA-DR dependent manner through class switching and somatic hypermutation (100). B cells and neutrophils together bring about a vicious circle. B-cell-derived IL-8 recruits neutrophils into the synovium for activation. In turn, primed neutrophils activate B cells in the synovium by continuously releasing cytokines, such as BAFF and IL-21 (101).

To date, there is no literature about the impact of RNA modification on immune cells (B cells) in synovium, especially pseudouridine. However, only a few studies have examined the impact of RNA modification on B cells in the cancer micro-environment. Kang and colleagues elucidated stage-dependent reliance on METTL3-mediated m6A of B cell development (95). They demonstrated that in the pro-B stage, METTL3 deficiency would affect the development and function of B Cells and the fibrogenic activity of B cells in liver fibrosis (102).

New research has indicated that METTL14-mediated m6A modification plays a crucial part in the response of gastric cancer B cells (101). Further demonstrated that the Mettl14/m6A/Ythdf2 axis inhibits negative regulators’ expression through mRNA decay, which is mandatory for the selective favour of GC B cells. Furthermore, inhibition of gene expression by M6A modification guides suitable determination of B cell fate at the beginning of the adaptive immune response. For example, METTL3 mediated m6A modification is required for maintaining GC stability through the function of different m6A readers (103, 104). The m6A binder IGF2BP3 is necessary to stabilise the expression of MYC mRNA and the target gene, while the m6A reader YTHDF2 acts indirectly to regulate the expression of oxidative phosphorylation. Grove et al. found YTHDF2 inhibits the genetic program of plasma cells and promotes the formation of GC at the initial stage of B cell immunological response (104, 105). It is worth noting that according to the analysis of CIBERSORT and TIMER databases, METTL16 (m^6^A “writer”) expression exhibits a significantly positive association with the invasion of B cells and CD8+T cells (106).

Chuanxi Yang and coworkers systematically analysed the prognostic and immunological roles of the ac4C regulator NAT10 in Pan-cancer (69). Contrasting with normal tissues, the expression of NAT10 significantly affects the prognosis of patients with pan-cancer. There is a positive association between NAT10 expression and infiltration of tumor immune cells, consisting of B cells, fibroblasts, CD8^+^ T cells, CD4^+^ T cells, macrophages, and neutrophils in LIHC. Therefore, it also represents a valuable research direction for tumor research.

## 5 Promising therapeutic strategies by targeting RNA modification

Up to now, little research has been done on RNA modification in the synovial microenvironment. Different enzymes affect RNA modification in various tissues at different stages of development, permitting the expression of genes related to the physiological functions of bones.

Therefore, it is useful to understand the intricate regulatory mechanism of RNA modification to target regulators for treating bone-related disorders. For instance, METTL3 facilitates the activation and inflammation of FLSs via the NF-κB signalling pathway (59, 107). Through the facilitation of M1 macrophage development, METTL3 stimulates osteogenic differentiation and migration of bone marrow mesenchymal stem cells (81). To ensure that a TFH transcriptional program is activated, METTL3 modifies m6A to stabilise Tcf7 transcripts (90). METTL3 makes it easier for RNA exosomes to find and process the 3’ end of SοGLT. This stops chromosomal translocations and encourages class switch recombination (CSR) (108). According to previous studies, METTL3 has emerged as a unique therapeutic target for bone-related disorders. Based on the virtual screening of ZINC and Drug Bank 4.0 databases, 11 compounds specific inhibitors of METTL3 have been identified, such as chidamide, STM2457 and UZH1a (109).

Similarly, a series of FTO inhibitors have been developed based on artificial intelligence methods or obtained from high-throughput screening in old drug libraries. As many as 14 inhibitors are known, mainly showing anti-cancer solid properties. In addition, potent ALKBH5 inhibitors were identified from a compound library (110, 111).

Most traditional medicines contain natural products and could be used as a chemical library for developing RNA modulators targeted therapeutic drugs. Curcumin and resveratrol are natural phenolic compounds with antioxidant and anti-inflammatory properties widely used to improve the pain and inflammation of joint diseases (112–114). In 2019, Gan et al. published a new finding that combining curcumin and resveratrol effectively improved intestinal mucosal integrity and growth performance by reducing m6A as evidenced by increasing YTHDF2 in the ileum (112, 115). This work represented a significant step forward by exploring the biological activities of traditional medicine targeting these modulators.

Although these potential candidate drugs are mentioned above, there is still a long way to go before they are used in clinical practice. Poor target specificity, therapeutics, safety, pharmacokinetics and other obstacles significantly hinder the advancement of RNA modification inhibitors and activators as therapeutic agents, specifically for the synovial micro-environment, which are bottlenecks from theory to clinical practice. Therefore, we urgently need to deeply investigate the crucial coupling effects among several types of cells in the subchondral bone micro-environment and discern the new mechanism in this process to formulate effective treatment strategies for RA by regulating the stability of the subchondral bone micro-environment.

## 6 Conclusion and perspectives

The current consensus is that as many as 170 post-transcriptional RNA modifications are known. With the renovation of various sequencing technologies, including progress in high-throughput NGS technology, the upgrading of liquid chromatography sensitivity, and other sequencing technologies, people have a deeper understanding of the totality level of RNA methylation.

Although substantial progress has been made in the synovial micro-environment, numerous problems remain to be solved, and multiple research gaps need to be filled to elaborate further on the effects and mechanisms of RNA modification on osteoimmunology or autoimmunity.

First, do RNA modifications control the biological processes of an immune cell? Do they serve as the main mediators or are they merely supporting actors? Therefore, how much can it play in treating immune-related diseases by correcting abnormal RNA modification? This will be the primary problem that needs to be eliminated when designing targeted therapeutic drugs.

Secondly, many RNA modifications are reversible, dynamically regulated and complex. However, how to achieve this dynamic balance precisely needs more exploration.

Thirdly, their functions as RNA modifications are executed by various enzymes (writers, erasers, and readers), which affect different downstream target RNAs. However, quite a few studies are needed to clearly clarify the complicated mechanisms of these regulators’ normal or abnormal action. Research on influencing factors of these regulators represents a new branch of research on RNA modification.

Fourthly, owing to the current research hotspot is on a few changes (such as m6A) and immune cells (such as macrophages), while the interaction between other modifications and immune cells is rarely studied. Therefore, it is urgent to further explore the role of RNA modifications in various immune cells. Immune cells interact, and various immune and inflammatory-related factors and signalling pathways are regulated by RNA modifications. These constitute a complex regulatory network, which demanded considerable effort from researchers.

Above all, targeted RNA modifications have a long way to go as a therapeutic method for immune-related diseases, especially inflammatory rheumatic diseases. At present, there is no successful clinical application report. For this reason, RNA modification and its regulation mechanism are the bottlenecks of research progress in this field.

We over viewed six RNA modifications, including m^6^A, m^1^A, m^5^C, m^7^G, ac4C, Ψ with their critical roles in immune cells and synovial homeostasis. Different RNA-modifying enzymes perform RNA modification functions in a variety of ways and participate in RNA regulation processes, including production, transport, stability, and metabolism. Considering these molecular roles, RNA modification mediates numerous biological functions of immune cells, including activation, differentiation, development, polarisation and migration, regulates synovial homeostasis, and promotes the pathogenesis of autoimmune inflammatory rheumatic diseases.

For a long time, m^6^A has been the star of RNA modification research, especially in the research branch of the immune micro-environment, because it has relatively sufficient background knowledge and mature research technology as support. For inflammatory arthritis, RNA modifications are primarily investigated in FLS, T cells in synovium and macrophage-related immune processes. In addition, the global pandemic of COVID-19 and antiviral immunity have also remained an interesting research topic for analysis of RNA modifications.

To sum up, this review provides a glimpse into the role of RNA modification and synovial homeostasis, which will provide valuable knowledge overview and novel ideas for investigators in the field of inflammatory rheumatic diseases.

## Glossary

ALKBH5: Alk B homolog 5
ALYREF: Aly/REF nuclear export factor
ATG7: Autophagy-related 7
DKC1: Dyskerin Pseudouridine Synthase 1
ELAVL1: ELAV Like RNA Binding Protein 1
FCγR: Fc gamma receptors R
FMRP: Fragile X mental retardation protein
FTO: Fat mass and obesity-associated protein
HAKAI: Homo sapiens Cbl proto-oncogene like 1
HLA-DR: Human leukocyte antigen DR
IGF2BP3: Insulin-like growth factor-2 messenger RNA-binding protein 3
LARP1: La-related protein 1
LIHC: Liver hepatocellular carcinoma
METTL3: Methyltransferase-like 3
METTL14: Methyltransferase like 14
NAT10: N-acetyltransferase 10
Osterix: Osteoblast-specific transcription factor
PUSs: Pseudouridine synthetases
PPAR-γ: Peroxisome proliferator-activated receptor gamma
RBM15: Homo sapiens RNA binding motif protein 15
Runx2: RUNX Family Transcription Factor 2
RPUSD1-4: Homo sapiens RNA pseudouridylate synthase domain containing 1-4
STAT1/5: Signal transducers and activators of transcription 1/5
SOCS: Suppressor of cytokine signalling
SμGLT: Immunoglobulin heavy chain (IgH) locus-associated G-rich long noncoding RNA
TET1: Ten-eleven translocation 1
THUMPD1: THUMP Domain Containing 1
TGM2: Transglutaminase type 2
TRMT10C: Human tRNA methyltransferase 10 homolog C
VEGF: Vascular endothelial-derived growth factor
VIRMA: Vir Like M^6^A Methyltransferase Associated
WTAP: Wilms tumor 1 associated protein
YTHDF1: YTH domain family 1
YTHDF2: YTH domain family 2
YTHDF3: YTH domain family 3
ZC3H13: Zinc Finger CCCH-Type Containing 13
